# Mpox Outbreak: A Call for Urgent Action and Improved Response Strategies

**DOI:** 10.1002/hsr2.70940

**Published:** 2025-07-07

**Authors:** Majani Edward, Grace Gwanafyo, Elizabeth A. Kimambo, Daniel Basaya

**Affiliations:** ^1^ Department of Public Health St. Francis University College of Health and Allied Sciences Ifakara Tanzania

**Keywords:** global threat, mpox, response strategies, surveillance, urgent call

## Abstract

**Introduction:**

The resurgence of mpox presents a significant global public health challenge, especially in non‐endemic regions. Following its initial global spread in 2022, the current outbreak is driven by a newly emerged Clade I, exhibiting atypical clinical manifestations and altered transmission patterns. These changes, coupled with limited immunity in younger populations due to the absence of smallpox vaccination, underscore the severity. As of June 2024, nearly 100,000 cases have been documented worldwide, with Africa experiencing the majority of new cases, highlighting critical gaps in global health preparedness.

**Methodology:**

This article provides an updated analysis of recent mpox cases, exploring the distinct epidemiological and clinical challenges of the current outbreak. It examines transmission patterns, affected demographics, and obstacles like underreporting to understand the evolving crisis.

**Results:**

The current outbreak is primarily centered in the Democratic Republic of Congo but poses global transmission risks. The dominance of the more severe Clade I and a significant shift towards human‐to‐human transmission, predominantly through sexual contact (83.3% of cases in males aged 29–41), characterize its epidemiology. Inadequate surveillance and underreporting, particularly in low‐resource settings, obscure the true extent of the outbreak. The analysis reveals the urgent need for a coordinated, multisectoral global response.

**Conclusion:**

Mpox outbreak underscores critical deficiencies in global health preparedness. An effective response requires an evidence‐based, coordinated approach, integrating technical, social, and financial resources internationally and locally. Key recommendations include strengthening surveillance, improving diagnostics, enhancing equitable vaccine accessibility, and implementing robust anti‐stigma initiatives at all levels. An inclusive, multisectoral collaboration between governments, international organizations, and community leaders is crucial to mitigate the public health, social, and economic impacts of this persistent outbreak.

## 1 Introduction

Mpox, historically known as monkeypox, was initially identified in humans in 1970 in the Democratic Republic of Congo (DRC) [1]. The disease has since been largely confined to Central and West Africa [2]. However, a significant shift occurred in May 2022, when mpox cases began spreading across multiple non‐endemic regions, eventually leading the World Health Organization (WHO) to declare it a Public Health Emergency of International Concern (PHEIC) on June 23, 2022 [1, 3]. By the end of October 2022, 76,871 cases had been reported across 19 non‐endemic countries, highlighting the rapid and unexpected global spread of the virus [3]. The mpox virus comprises two major clades: Clade I (Congo Basin) and Clade II (West African). Historically, Clade I is known for causing more severe symptoms and higher mortality rates, while Clade II is associated with milder infections [3]. However, the current outbreak has seen the emergence of a new clade, exhibiting atypical clinical symptoms, such as dermatological, neurological, and respiratory manifestations. These unusual symptoms and recent transmission patterns—often linked to close‐contact behaviors, including sexual contact in men who have sex with men—represent a significant epidemiological shift [4]. The outbreak also reflects the impact of limited immunity due to the absence of smallpox vaccination coverage in younger populations [5].

Between January 1, 2024, and June 30, 2024, the WHO documented 99,176 cases across 116 countries. June 2024 alone saw 934 new cases, with Africa accounting for 61% of these cases, followed by 19% in the Americas and 11% in Europe [6]. This resurgence highlights critical gaps in global health preparedness, with the persistence and evolution of mpox underscoring the need for enhanced surveillance, diagnostic innovations, and international cooperation to prevent further spread.

## 2 Current Situation and Key Challenges

The current mpox outbreak presents unique challenges that demand urgent attention. The DRC remains the epicenter, contributing 96% of confirmed cases, with neighboring countries such as Rwanda, Uganda, Burundi, and Kenya now reporting their first cases [7]. Although Africa is the most affected, cases in the Americas, Europe, and other regions indicate global transmission risks [4, 8]. Epidemiologically, the 2024 outbreak has been dominated by Clade 1, known for its higher severity compared to Clade 2. This clade's rapid spread and more severe clinical outcomes have heightened public health concerns [3]. In contrast to earlier outbreaks primarily linked to animal contact, recent cases are increasingly driven by human‐to‐human transmission, especially through sexual contact [7]. A significant 96.4% of confirmed cases are in males aged 29–41, with 83.3% of transmissions occurring via sexual contact. While transmission is linked to a sexual contact, it can still affect a diverse population. The outbreak has also affected children, particularly in the Americas, though they constitute a smaller proportion of cases. The limited immunity from past smallpox vaccinations in older adults further complicates the situation, leaving many populations vulnerable [6]. Underreporting remains a significant obstacle to effective outbreak management. Surveillance systems, particularly in low‐resource settings, are inadequate, leading to gaps in data collection and underestimation of case numbers. Asymptomatic or mildly symptomatic cases often go undetected, contributing to the silent spread of the virus. The focus on symptomatic cases also limits our understanding of the true extent of the outbreak, emphasizing the need for more robust and comprehensive surveillance mechanisms [9].

## 3 Call to Action

The ongoing mpox outbreak demands a swift and decisive global response. Current control measures have proven insufficient, necessitating a reassessment and revision of strategies [9]. A critical priority is the allocation of more resources, including increased funding, expanded vaccine access, and the deployment of trained personnel, especially in regions with limited healthcare infrastructure [10]. Ensuring equitable vaccine distribution is essential to protect high‐risk populations and prevent further spread. Enhanced global coordination is vital to harmonizing response efforts across countries [11]. This includes standardized guidelines for case management, improved communication channels, and stronger international partnerships. Surveillance systems must be strengthened to capture more accurate data, including asymptomatic cases, which are crucial for controlling transmission. The global community should also prioritize the development of targeted interventions for populations disproportionately affected by the outbreak, such as sex workers, high‐risk adults, and vulnerable groups in endemic regions [12]. Additionally, public health messaging should focus on reducing stigma and discrimination associated with mpox, particularly in communities where transmission is linked to sexual behavior [5]. This stigma, which parallels that seen during the early HIV epidemic, can create barriers to accessing healthcare and adhering to preventive measures. Effective interventions must occur at multiple levels—policy, healthcare organization, and community—to reduce discrimination and promote equitable care. To avoid stigmatizing specific populations, public health messaging should focus on promoting understanding of mpox transmission and symptoms without reinforcing biases. Community‐based education campaigns, inclusive messaging, and policy‐level initiatives aimed at protecting affected populations are critical in fostering cooperation with public health measures [13].

Addressing these social determinants of health is essential to improving public trust and compliance with outbreak response strategies [14].

## 4 Conclusion

The 2024 mpox outbreak has underscored critical deficiencies in global health preparedness and response. Addressing these gaps requires an evidence‐based, coordinated approach that combines technical, social, and financial resources across international and local levels. Strengthening surveillance, improving diagnostics, enhancing vaccine accessibility, and fostering anti‐stigma initiatives are necessary to protect vulnerable populations and prevent future resurgences. An inclusive, multisectoral response that leverages collaboration between governments, international organizations, and community leaders is essential to mitigate the public health, social, and economic impacts of this outbreak.

## Author Contributions

**Majani Edward:** conceptualization, writing – original draft, writing – review and editing, supervision, and investigation. **Grace Gwanafyo:** writing – original draft and investigation. **Eliza Kimambo:** writing – original draft and investigation. **Daniel Basaya:** investigation and writing – original draft.

## Ethics Statement

The authors have nothing to report.

## Consent

The authors have nothing to report.

## Conflicts of Interest

The authors declare no conflicts of interest.

## Transparency Statement

The lead author, Majani Edward, affirms that this manuscript is an honest, accurate, and transparent account of the study being reported; that no important aspects of the study have been omitted; and that any discrepancies from the study as planned (and, if relevant, registered) have been explained.

## Data Availability

The data presented in this article are derived from publicly available reports and analyses from organizations such as the World Health Organization (WHO) and other referenced epidemiological sources. All specific data points and statistics mentioned are attributed to their original publications within the text. No new, primary, or proprietary data sets were generated or analyzed for this review. Further details on the referenced data can be found in the cited literature.
